# DHEAS Elevation as the Early Biochemical Abnormality in ACTH-Secreting Pituitary Microadenomas in Children: Two Case Reports

**DOI:** 10.2174/0118715303421171260109070559

**Published:** 2026-04-22

**Authors:** Qiting Zhang, Yiling Cui, Wenjun Long, Ling Hou, Yanqin Ying, Xiaoping Luo

**Affiliations:** 1 Department of Pediatrics, Tongji Medical College, Tongji Hospital, Huazhong University of Science and Technology, Wuhan 430030, China;; 2 Hubei Key Laboratory of Pediatric Genetic Metabolic and Endocrine Rare Diseases, Wuhan 430030, China;; 3 Hubei Provincial Clinical Research Center for Child Growth, Development, and Metabolic Diseases, Wuhan 430030, China

**Keywords:** ACTH-secreting pituitary microadenomas, dehydroepiandrosterone sulfate, androstenedione, biochemical abnormalities, Cushing's disease, MRI

## Abstract

**Introduction:**

ACTH-secreting pituitary microadenomas are associated with insidious onset in children and atypical early clinical manifestations, making them highly susceptible to misdiagnosis and underdiagnosis. They are characterized by elevated ACTH and cortisol levels with a disrupted circadian rhythm. We reported two pediatric cases that showed significant elevation of Dehydroepiandrosterone Sulfate (DHEAS) and Androstenedione (Ad) in the early stage, while ACTH and cortisol were in the normal range or mildly abnormal.

**Case Presentation:**

Case 1, a 13-year-old male, was admitted to the hospital with two years of growth retardation. The initial pituitary Magnetic Resonance Imaging (MRI) was normal. After one year, the patient developed Cushing's Syndrome (CS), and a repeat MRI suggested a pituitary microadenoma. Case 2, a 13.1-year-old female who had experienced excessive weight gain for two years and amenorrhea for 11 months. The child presented with CS initially, and the MRI revealed a pituitary microadenoma. Both of them were pathologically confirmed as ACTH-secreting pituitary microadenomas after Transsphenoidal Surgery (TSS).

**Conclusion:**

This report demonstrates that in cases with a significant DHEAS elevation, even with near-normal ACTH/cortisol levels and negative pituitary MRI, high clinical suspicion for ACTH-secreting microadenomas should be maintained, warranting close follow-up and further evaluation.

## 1 INTRODUCTION

Pituitary microadenomas refer to tumors that originate from the hormone-secreting cells of the anterior pituitary lobe with a diameter of < 10 mm [1]. In children, they are often discovered incidentally during clinical examinations [2, 3], and most are non-functional adenomas [4, 5], which brings challenges to clinical diagnosis and treatment [6].

ACTH-secreting pituitary microadenomas are very rare in children and can secrete excessive amounts of ACTH, causing hypercortisolism, also known as Cushing’s Disease (CD). Compared with adult patients, pediatric presentations may be subtle [7]. This condition is clinically characterized by growth failure (90–100%), facial changes (70–100%), rapid weight gain (92–98%), and abnormalities in sexual development [8]. The main biochemical features include an abnormal circadian rhythm of serum cortisol, elevated cortisol levels at midnight, and increased 24-h urinary free cortisol excretion.

Herein, we report the clinical data of two children with ACTH-secreting pituitary microadenomas who had prominent biochemical abnormalities in terms of serum DHEAS and Ad in the early stages of disease progression.

## 2 CASE PRESENTATION

### 2.1 Case 1

A 13-year-old male was admitted to the hospital with two years of growth retardation. His height was 144 cm (< 3rd percentile), and his blood pressure was 146/78 mmHg. There were no clinical signs of CS. Pubertal development had not begun. Bone age was assessed at 12.5 years according to the Greulich and Pyle atlas.

The following investigations were completed during the initial hospitalization. Tumor markers (AFP, CEA, CA-125, CA19-9, CA15-3, NSE, and β-hCG) were all negative. The GH stimulation test showed a peak concentration of 5.84 ng/mL in 60 minutes. To identify the etiology of hypertension, we investigated the renin-angiotensin-aldosterone system and performed an ultrasound; no abnormalities were identified. Analysis of the adrenal hormones revealed extremely high serum levels of DHEAS at 22,900 nmol/L and Ad at 13.70 nmol/L. In contrast, the serum levels of ACTH and cortisol were within the normal range (Table **1**). Adrenal Computed Tomography (CT) and pituitary MRI were negative. No relevant pathogenic mutations were identified in the adrenal-targeted gene panel or in the subsequent whole-exome sequencing. At this juncture, with life-threatening pathologies ruled out and the patient clinically stable, the parents opted for discharge despite the unexplained elevated DHEAS.

It was one year later that the patient returned to our hospital with manifestations of moon facies, facial plethora, and mild acneiform rash. Analysis of adrenal hormones was performed (ACTH at 8 am: 56.0 pg/mL; cortisol: 20.2 µg/dL; DHEAS> 27100.00 nmol/L; Ad: 11.30 nmol/L). There was a disturbed circadian rhythm for ACTH. The 24-h urinary 17-hydroxycorticosteroids (4.79 mg/24 h) were elevated. Neither low nor high-dose dexamethasone suppression tests were suppressed (Table **2**). MRI suggested a possible pituitary microadenoma. Surgery was recommended, but his parents declined.

The child's clinical symptoms persisted for four months after discharge, and the serum levels of DHEAS and Ad remained high. Therefore, Transsphenoidal Surgery (TSS) was performed, and the pathological analysis was consistent with ACTH-secreting pituitary microadenomas. Postoperative evaluation revealed secondary adrenal insufficiency (ACTH at 8 am: 2.8 pg/mL; cortisol: <0.4 µg/dL; DHEAS: 839 nmol/L; Ad: 0.34 nmol/L) and transient GH deficiency, with other pituitary axes remaining intact. After over two months of hydrocortisone acetate replacement, ACTH and cortisol levels normalized. During follow-up, the patient underwent normal puberty at 13-14 years of age and exhibited significant catch-up growth, with the height SDS improving from -2.09 to -0.57.

### 2.2 Case 2

A 13.1-year-old female was admitted to our hospital with the symptoms of excessive weight gain for two years and amenorrhea for 11 months. Her height was 159 cm, and her BMI was 28.16 kg/m^2^ (>95th percentile). Her blood pressure was 134/85 mmHg, and she was centripetally obese, with purple lines, hirsutism, and acne.

The patient fulfilled four criteria for metabolic syndrome by having abdominal obesity, disturbed glucose metabolism, hyperlipidemia, and hypertension. To assess the patient's hypothalamic-pituitary axis function, we performed a gonadotropin-releasing hormone stimulation test. It revealed a peak LH level of 7.72 IU/L at 30 minutes (baseline LH: 0.11 IU/L), with an LH/FSH ratio of 0.895.

Analysis of adrenal hormones revealed extremely high serum levels of DHEAS (20600.00 nmol/L) and Ad (23.4 nmol/L), while ACTH and cortisol were mildly increased (Table **1**). The 24-h urinary 17-OHCS was elevated. Following the LDDST, serum cortisol failed to suppress. HDDST shows a cortisol suppression rate close to 50% (Table **2**). MRI revealed a pituitary microadenoma. Given the absence of visual field deficits or impaired visual acuity, there was no clear indication for TSS. The management strategy consisted of surveillance, including follow-up with endocrinology and neurosurgery, and a recommended pituitary MRI with contrast for further characterization. After discharge, she received treatment with metformin, levothyroxine sodium, potassium chloride tablets, and febuxostat for significant hyperuricemia (571.2 umol/L).

**Fig. (1):**
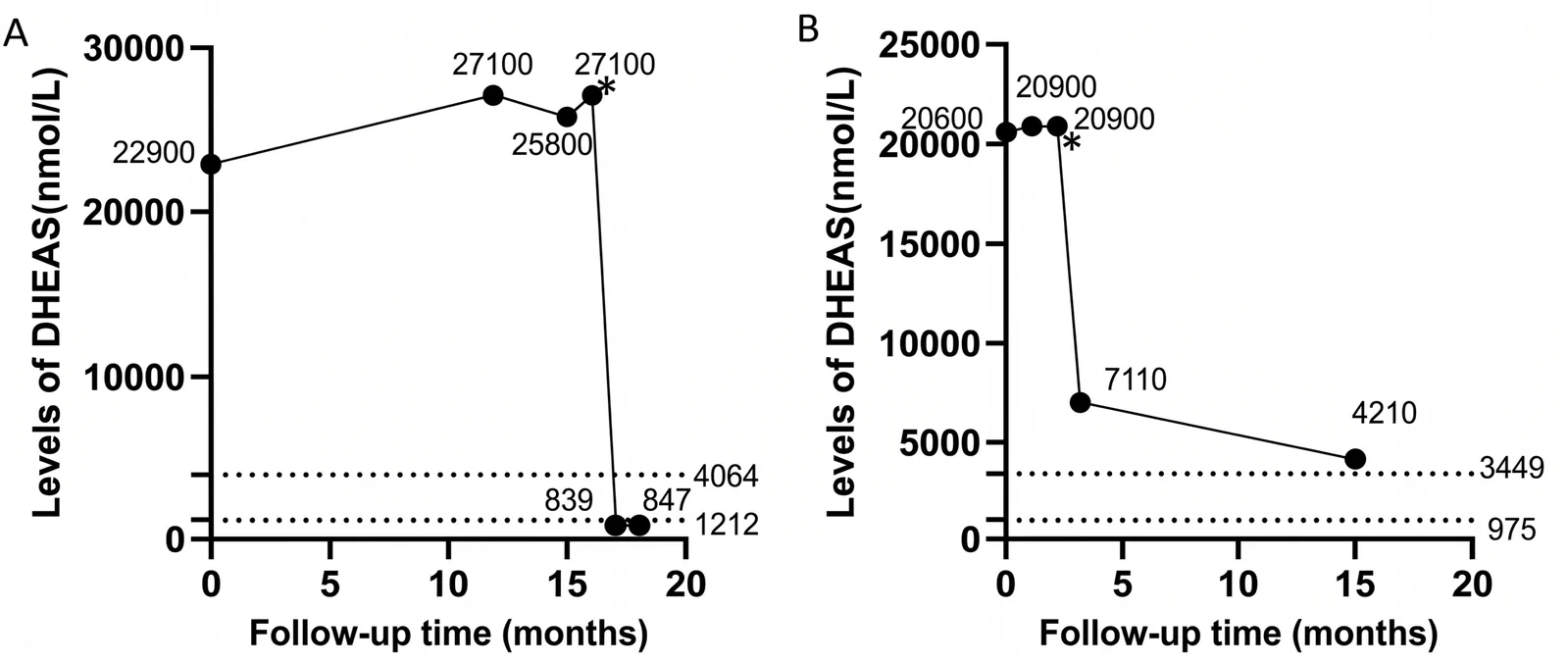
Changes of DHEAS during disease progression for (**A**) Case 1 and (**B**) Case 2. (* Indicates preoperative status).

Two months later, the patient had experienced a progression of symptoms and persistently elevated adrenal hormones (Fig. **1**). Therefore, the patient underwent TSS; our pathological findings were consistent with an ACTH-secreting pituitary microadenoma. Postoperative pituitary evaluation revealed transient GH deficiency. Since cortisol levels (187.8 µg/L; reference range: 60.2-184 µg/L) remained elevated, hydrocortisone replacement was not initiated. At the one-month follow-up, a significant reduction in adrenal hormones was observed (ACTH at 8 am: 47.6 pg/mL; cortisol: 26.6 µg/dL; DHEAS: 7110 nmol/L; Ad: 11.90 nmol/L). Due to the recurrence of the tumour about one year after the initial surgery, the patient underwent a second operation at an outside hospital and was subsequently lost to follow-up.

## 3 DISCUSSION

DHEAS is the most abundant steroid hormone in the human circulatory system; the levels of this hormone are regulated by ACTH, and their circadian rhythms remain parallel [9]. DHEAS is known to be influenced by a number of factors. Elevated levels of DHEAS can be detected in obese and hypertensive patients [10]. Furthermore, DHEAS can be significantly elevated in certain congenital adrenocortical hyperplasias (21-hydroxylase deficiency, 11β-hydroxylase deficiency [10]) and in androgen-secreting adrenal tumours [11].

**Fig. (2):**
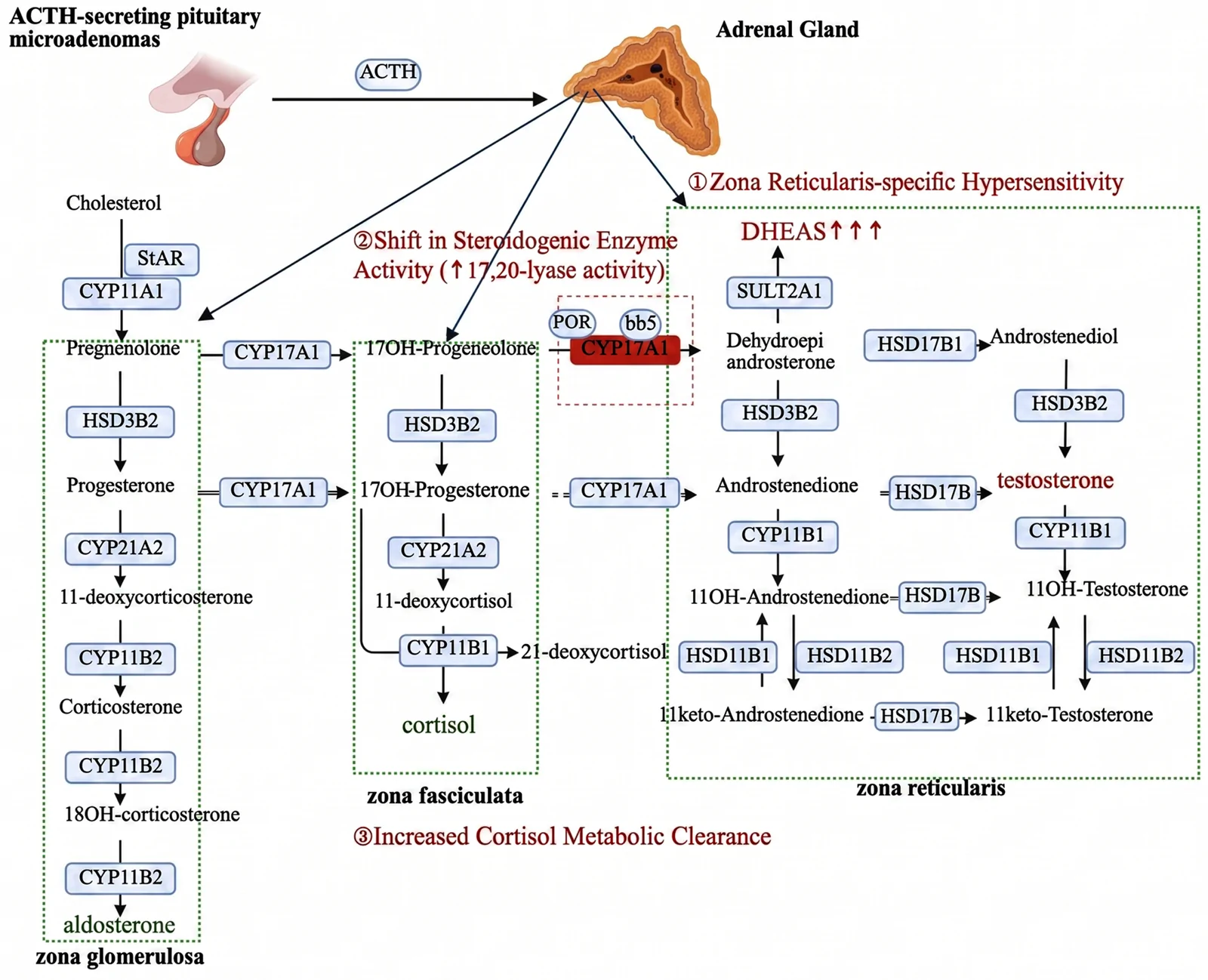
Proposed mechanisms for the disproportionate elevation of DHEAS relative to cortisol in early-stage ACTH-secreting microadenoma. **Abbreviations:** StAR: steroid acute regulatory protein; CYP: cytochrome P450; CYP11A1 (P450scc): cholesterol desmolase (cholesterol side-chain cleavage enzyme); HSD3B2: 3-hydroxysteroid dehydrogenase type 2 (3B-HSD2); CYP21A2 (P450c21): 21-hydroxylase; CYP11B1 (P450c11B): 11ß-hydroxylase; CYP11B2 (P450c11AS): aldosterone synthase; CYP17A1 (P450c17): 17c-hydroxylase/17,20-lyase; HSD11B1/HSD11B2: 11ß-hydroxysteroid dehydrogenase types 1 and 2; HSD17B: 17ß-hydroxysteroid dehydrogenase; SULT2A1: DHEA sulfotransferase; SRD5A: 5c-reductase.

The significant elevation of DHEAS in the early stages of ACTH-secreting pituitary microadenomas has not been reported previously. Herein, we report the cases of two patients who both showed significant elevation of DHEAS in the early stage when the ACTH and cortisol secretion rhythms had yet to change, thus suggesting that a significant elevation of DHEAS may be used as an early marker of ACTH-secreting pituitary microadenomas. While the precise mechanisms warrant further investigation, we propose a model wherein the dissociation between adrenal androgen and cortisol secretion in response to minimal ACTH stimulation may result from zona reticularis-specific hypersensitivity, dysregulation of steroidogenic enzymes, and an increased cortisol metabolic clearance rate (Fig. **2**).

MRI is the method of choice for the detection of ACTH-secreting pituitary microadenomas [12]. However, in the latest and largest paediatric series, it was reported to detect only 16%–71% of adenomas [13]. In our study, initial pituitary MRI scans found no abnormalities in case 1, although the patient had already exhibited a significant increase in DHEAS, which suggested that DHEAS may be more sensitive than MRI. Since this article only reports two cases, further validation with a larger sample size is urgently needed.

Bilateral Inferior Petrosal Sinus Sampling (BIPSS) has been proposed to confirm ACTH-secreting microadenomas in MRI-negative cases with high clinical suspicion [14]. However, its invasiveness restricted widespread use. Emerging techniques, such as high-resolution 3T-MRI with 3D-spoiled gradient-echo sequences and molecular PET/CT or PET/MRI, may improve diagnostic efficiency [15], but these remain limited to specialized centers. In contrast, DHEAS may be a promising indicator for early diagnosis of the disease.

The treatment of choice for patients with ACTH-secreting microadenomas is TSS [16]. The existing literature reports that 98% of patients can achieve postoperative remission [17]. Radiation therapy with interim medical therapy and bilateral adrenalectomy are key options for patients with persistent or recurrent CD after TSS [18]. Recent advances in pharmacotherapy and refined neurosurgical techniques for Cushing's disease have demonstrated significant improvements in clinical outcomes, effectively mitigating the disease burden [19-21].

## CONCLUSION

This study reports two cases of ACTH-secreting pituitary microadenomas with early biochemical manifestations characterized by significantly elevated DHEAS and Ad. The diagnostic challenge stems from non-specific clinical presentations, near-normal ACTH-cortisol axis levels, and negative pituitary MRI findings, suggesting DHEAS as a potential early biomarker. However, the limited sample size necessitates cohort expansion for validation and further investigation into the underlying mechanisms driving aberrant DHEAS secretion in pituitary microadenomas.

## Figures and Tables

**Table 1:** Clinical data of two cases on admission.

-	**Case 1**		**Case 2**		-
Test	Value	Reference	Value	Reference	Units
Age	13	-	13.1	-	Years
Gender	Male	-	Female	-	-
BP	146/78	<130/80	134/85	<130/80	mmHg
Laboratory test					
TSH	0.540	0.6-4.5	0.23	0.6-4.5	mIU/L
TT3	1.04	1.21-2.66	0.41	1.21-2.66	nmol/L
TT4	87.5	67-163	33.1	67-163	nmol/L
FT4	1.1	0.8-1.9	0.80	0.8-1.9	ng/dl
Renin (Standing/lying)	8.4/3.7	4.4-46.1/2.8-39.9	1.4	4.4-46.1/2.8-39.9	uIU/mL
Aldosterone (Standing/lying)	61.4/43	0-353.0/0-236.0	38.6	0-353.0/0-236.0	pg/mL
Adrenaline	<0.07	≤0.21	-	-	nmol/L
NE	0.28	≤0.59	-	-	nmol/L
K	4.21	3.50-5.10	3.25	3.50-5.10	mmol/L
Na	141.2	136-145	140.9	136-145	mmol/L
Cortisol(8am)	17.0	6.7–22.6	57.8	6.7–22.6	ug/dL
ACTH(8am)	42.8	7.2–63.3	79.0	7.2–63.3	pg/mL
DHEAS	22,900	2,638±1,426	20600	2212±1237	nmol/L
Ad	13.70	2.49±1.11	23.4	2.86±1.65	nmol/L
Imaging					
MRI	(-)		(+)		-
Adrenal CT	(-)		(-)		-

**Abbreviations:** NE, Norepinephrine; ACTH, Adrenocorticotropic Hormone; AD, Androstenedione.

**Table 2:** Results of low and high-dose dexamethasone suppression tests for two cases.

-	**Case 1**			**Case 2**		
	Pre-test	LDDST	HDDST	Pre-test	LDDST	HDDST
Cortisol (6.7–22.6 µg/dL)	20.2	16.9	19.5	57.8	44.8	28.5
24-h 17-OHCS (2-10 mg/24 h)	14.79	12.90	15.4	22.57	22.24	11.8
24-h 17-KS (6–25 mg/24 h)	13.43	17.70	26.42	8.89	7.20	7.20

**Abbreviations:** 17-OHCS: 17-Hydroxycorticosteroids; 17-KS: 17-ketosteroids; HDDST: high-dose dexamethasone suppression test; LDDST: low-dose dexamethasone suppression tests.

## Data Availability

All data generated or analyzed during this study are included in this published article.

## LIST OF ABBREVIATIONS

DHEAS: Dehydroepiandrosterone Sulfate
Ad: Androstenedione
ACTH: Adrenocorticotropic Hormone
BIPSS: Bilateral Inferior Petrosal Sinus Blood Sampling
CD: Cushing's Disease
CS: Cushing's Syndrome

## STANDARDS OF REPORTING

CARE guidelines were followed.
